# Protective role of murine norovirus against *Pseudomonas aeruginosa* acute pneumonia

**DOI:** 10.1186/s13567-015-0239-3

**Published:** 2015-09-04

**Authors:** Marion Thépaut, Teddy Grandjean, Didier Hober, Pierre-Emmanuel Lobert, Perrine Bortolotti, Karine Faure, Rodrigue Dessein, Eric Kipnis, Benoit Guery

**Affiliations:** EA 7366: Pseudomonas aeruginosa Host-Pathogen Translational Research Group, UDSL, Université Lille Nord de France, 59045 Lille, France; Laboratoire de virologie EA3610, Faculté de Médecine, CHRU, Université Lille 2, 59037 Lille, France

## Abstract

The murine norovirus (MNV) is a recently discovered mouse pathogen, representing the most common contaminant in laboratory mouse colonies. Nevertheless, the effects of MNV infection on biomedical research are still unclear. We tested the hypothesis that MNV infection could alter immune response in mice with acute lung infection. Here we report that co-infection with MNV increases survival of mice with *Pseudomonas aeruginosa* acute lung injury and decreases in vivo production of pro-inflammatory cytokines. Our results suggest that MNV infection can deeply modify the parameters studied in conventional models of infection and lead to false conclusions in experimental models.

## Introduction

The first murine norovirus, MNV1, was isolated in 2003 from the brain of an immunocompromised mouse lacking recombination-activating gene two and signal transducer and activator of transcription one (RAG2/STAT1^-/-^) [1]. Murine noroviruses are non-enveloped, positive-strand RNA viruses that belong to the Norovirus genus in the *Caliciviridae* family. This virus is related to the human norovirus which is estimated to be responsible for up to 90% of nonbacterial epidemic gastroenteritis worldwide [2]. Like human norovirus, many strains of MNV have been isolated and biological diversity among MNV strains has also been reported [3-7]. Strains can either be rapidly cleared in wild type animals like MNV-1 CW1, CW3, and WU11, while CR1, CR3, CR6, CR7 and S99 have been published to be persistent [4,5].

Today, murine norovirus is the most prevalent virus in research mouse colonies [8]. In North America, 22.1% of 12,639 mouse sera were positive for anti-MNV-1 antibodies [9]. This high prevalence was confirmed by a serological survey in Europe [10]. Similar prevalence rates were observed in Japan and South Korea after serological or RT-PCR analysis of murine samples [11-13]. This worldwide high prevalence provides a tremendous potential for this virus to interfere with mouse models of diseases. However, the effects of MNV infection on biomedical research are still unclear.

Some studies showed that norovirus had no effect on specific animal models. Hensley et al. have shown that murine norovirus CR6 infection had no significant effect on adaptive immunity to vaccinia virus or influenza A virus [14]. Similarly, transient MNV 1 or persistent MNV-4 norovirus infection did not alter the pathology of *Salmonella typhimurium* induced intestinal inflammation and fibrosis in mice [15]. In another model, infection with murine norovirus 4 did not alter helicobacter-induced inflammatory bowel disease in interleukin 10^-/-^ mice [16].

However, several other studies showed potential consequences of norovirus infection. Lencioni et al. found that MNV infection could accelerate bacteria-induced inflammatory bowel disease progression in Mdr1a^-/-^ but not with Smad3^-/-^ mice [17]. Murine norovirus-1 was also reported to promote inflammation and mortality in mice superinfected with *Escherichia coli* [18].

To better understand the impact of MNV infection in animal models of bacterial pathogenicity, we investigated the effect of the MNV S99 strains on a *Pseudomonas aeruginosa* induced lung injury model in C57BL/6 mice. Co-infections may have unpredictable consequences with alterations of the host immune response and potentially mislead the investigator in the pathophysiological hypothesis based on the results. This study indicates that MNV-induced immunomodulation increases survival and decreases in vivo production of pro-inflammatory cytokines. These phenomena are a direct consequence of MNV infection and compromise the result obtained in *P. aeruginosa* studies.

## Materials and methods

### Mouse model

Wild-type C57BL/6 male mice, 8 to 10 weeks old, were purchased from Janvier laboratories. The mice had free access to a standard laboratory food diet in a half-day light cycle exposure and temperature. Mice were housed in a controlled Specified Pathogen Free (SPF) environment as determined by the FELASA recommendations, in either a static micro-isolator or individually ventilated cages. Vendor reports indicated mice were negative for murine norovirus. All animal studies were approved by the investigational review board of the Nord-Pas-de-Calais. All animal experiments were performed in an accredited establishment (N° B59-108) according to the governmental guidelines N°86/609/CEE.

### Cell lines

Raw264.7 cells (ATCC TIB-71) purchased from the European Collection of Cell Cultures (ECACC) (Sigma-Aldrich, L’isle d’Abeau Chesnes, France) were maintained in Dulbecco’s Modified Eagle Medium, high glucose, GlutaMAX™ Supplement, pyruvate (DMEM) supplemented with 10% heat inactivated fetal bovine serum (Gibco).

### Virus stocks and plate assays

All experiments were performed with Murine Norovirus S99 (Berlin/2006/DE) purchased from the Friedrich-Loeffler Institut (Greifswald-Insel Riems, Deutchland). Virus stocks were generated using Raw264.7 as described previously [19]. To generate a virus stock, viral suspensions were concentrated with Amicon Ultra-15, PLHK Ultracel-PL Membrane, 100 kDa (Merck Millipore). MNV titer was obtained by endpoint titration as described previously and expressed as TCID50/ml according to the Spearman-Kärber method [20,21]. The theoretical relationship between TCID50 and plaque forming units (PFU) is approximately 0.69 by applying the Poisson distribution [22].

### Bacterial strain

All experiments were performed with *Pseudomonas aeruginosa* strain CHA (CHA) provided by B. Toussaint (THeREx, Grenoble, France). A single colony was inoculated into Luria Bertani (LB) media and grown overnight at 37 °C with shaking. The day of the infection, a 1/40 dilution of the overnight culture was prepared and incubated 2 h at 37 °C with shaking. Bacteria were washed twice with sterile phosphate-buffered saline (PBS). The bacterial pellet was resuspended in PBS and optical density was measured at A_600_, the desired infectious dose was extrapolated from a standard growth curve. Bacterial suspensions and inoculum standardization were then determined based on spectrometry and verified by serial dilution and plating on bromocresol purple agar (BCP; bioMérieux, Marcy l’Étoile, France).

### Mouse infection pneumonia

C57BL6/J mice were infected by oral gavage with 1.10^7^ PFU of MNV S99, control mice received PBS. Seven days later, a pulmonary infection model was induced by intranasal instillation of *P. aeruginosa*. Mice were lightly anesthetized with inhaled sevoflurane (Forene Abbott, Queensborough, Kent, United Kingdom), after which 40 μL of the bacterial solution were administered intranasally representing a total of 2.10^6^ CFU/mouse (except for survival studies conducted with lethal inoculate of 5.10^6^ CFU/mouse). Control mice received 40 μL of PBS. All mice were sacrificed at 24 h (except for survival studies, mice were monitored on weight and clinical score 96 h post bacterial infection).

### Clinical score

The evolution of the disease score assessing the clinical appearance of the animals was evaluated on 4 items scored from 0 to 10 summarized in Table 1 (body temperature, appearance of the bristle, behaviour and weight loss; 0: healthy mice, 10 moribund mice). Clinical score = [(Fur score + temperature score + movement score) + (% weight loss/(−5))].

**Table 1:** **Clinical score evaluation**

**Score**	**Movement**	**Fur**	**Temperature**
0	Moving	Smooth	Normal
1	Low mobility		
2	Prostrate	Bristly	Cold

### Lung injury

Alveolo-capillary membrane permeability was evaluated by measuring Fluoresceine Isothiocyanate labeled (FITC)-albumin leakage from the vascular compartment to the alveolar-interstitial compartment, as previously described [23]. Briefly, mice were sacrificed after FITC-albumin injection, blood and bronchoalveolar lavage was collected. The fluorescence ratio measured in serum and bronchoalveolar lavage (Excitation: 487 nm, Emission: 520 nm; Mithras LB 940, Berthold Technologies, Bad Wildbad, Germany) reflects alveolo-capillary permeability.

### Bronchoalveolar lavage (BAL)

Lungs from each experimental group were washed with a total of 1.5 mL PBS. The recovered lavage fluid was centrifuged (200 *g* for 10 min), the cellular pellet was washed twice with PBS. BAL samples were frozen at −80 °C after collection for cytokine measurement. Cell counts were performed directly by optical microscopy. Cell monolayers were prepared with a cytocentrifugator and stained with a Wright-Giemsa stain. Cellular types were obtained by counting 200 cells/sample and expressing each type of cell as a percentage of the total number counted.

### Bacterial burden

Mouse lungs and spleens were homogenized in sterile containers with PBS. Lung and spleen homogenates were sequentially diluted and cultured on bromocresol purple agar plates for 24 h to assess bacterial load.

### Measurement of cytokines

Mouse cytokines, IL-6 and TNF-α were measured in culture or BAL supernatant using enzyme-linked immunosorbent Assay (ELISA) kits from Peprotech.

### Statistical analysis

Statistical analysis was carried out using Prism 5 software (Graph-Pad Software, San Diego CA). Values are expressed as mean ± SEM. Comparison groups were analysed with the nonparametric Mann–Whitney test. Survival curves were analysed using Log-rank (Mantel-Cox) test. Significance was accepted at *p < 0.05; ** *p* < 0.01; *** *p* < 0.001.

## Results

### Murine norovirus infection increases survival of mice with *P. aeruginosa* induced lung injury

**Figure 1:**
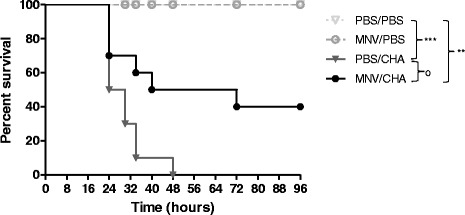
**Survival analysis.** WT mice were infected *per os* with 1.10^7^ PFU of MNV or PBS (*n* = 10/group). A week later, mice were infected with intranasal instillation of 5.10^6^ CFU of *P. aeruginosa* or PBS. Lethality was monitored for 96 h after *P. aeruginosa* infection. *, *P* < 0.05 for a comparison with the control PBS/PBS; **, *P* < 0.01 for a comparison with the control PBS/PBS; ***, *P* < 0.001 for a comparison with the control PBS/PBS; °*P* < 0.05 for a comparison with PBS/CHA.

To determine whether an established infection with MNV affected *P. aeruginosa* infection, survival was analysed for 96 h. After 7 days of colonisation with the virus, mice were infected by intranasal instillation of *P. aeruginosa*. No mortality was observed for two control groups PBS/PBS and MNV/PBS. A dose of 5.10^6^ CFU of *P. aeruginosa* induced 100% lethality in the PBS/CHA group. Infection with MNV before *P. aeruginosa* was associated to a 40% survival (Figure 1).

### Murine norovirus infection decreases *P. aeruginosa* induced lung injury and dissemination

**Figure 2:**
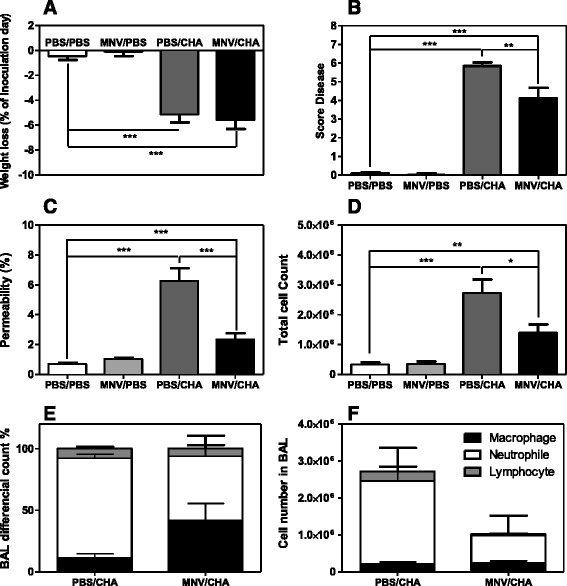
**Physiological and alveolar capillary evaluation.** WT mice were infected *per os* with 1.10^7^ PFU of MNV or PBS (*n* = 10/group). A week later, mice were infected with intranasal instillation of 2.10^6^ CFU of *P. aeruginosa* or PBS. Changes in body weight (**A**) and score diseases (**B**) were evaluated and mice were sacrificed at 24 h post-infection. Lung injury was assessed by alveolar capillary barrier permeability (**C**) and cells from Bronchoalveolar Lavage were counted (BAL) (**D–F**). *, *P* < 0.05; **, *P* < 0.01; ***, *P* < 0.001.

A dose of 2.10^6^ CFU of *P. aeruginosa* was inoculated and all the analyses were performed at 24 h after the bacterial challenge. Control groups, PBS/PBS and MNV/PBS, showed no body weight change and a normal clinical score. In *P. aeruginosa* infected mice, body weight significantly decreased and the clinical score significantly increased compared to the PBS/PBS group. MNV pre infection did not prevent body weight decrease but significantly reduced the clinical score compared to PBS/CHA mice (Figures 2A and B).

Regarding lung alveolar capillary permeability at 24 h, the efflux of the protein tracer in the MNV/PBS group was comparable to the control PBS/PBS group. In PBS/CHA infected animals, there was a significant increase of permeability compared to the PBS/PBS group. Mice pre-treated with MNV and further infected with *P. aeruginosa* presented a slight increase of permeability compared to control groups but significantly lower than the PBS/CHA group (Figure 2C).

Total cell counts in BAL were no different in PBS/PBS and MNV/PBS groups. *P. aeruginosa* infection, compared to the PBS/PBS group, induced a significant increase of the total cell count; MNV pre infection significantly decreased this number (Figure 2D). The differential analysis of the cells showed a decreased recruitment of neutrophils and lymphocytes in the MNV/CHA group compared to the PBS/CHA group (Figures 2E and F).

**Figure 3:**
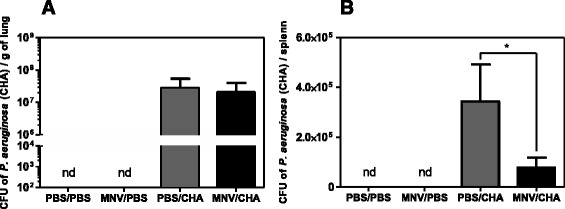
**Microbiological parameters.** WT mice were infected *per os* with 1.10^7^ PFU of MNV or PBS (*n* = 10/group). A week later, mice were infected with intranasal instillation of 2.10^6^ CFU of *P. aeruginosa* or PBS. Bacterial load in the lungs (**A**) and dissemination (**B**) assessed through spleen or cultured lung homogenate. *, *P* < 0.05; **, *P* < 0.01; ***, *P* < 0.001; nd: not detected.

Lung bacterial loads were not significantly different between PBS/CHA and MNV/CHA groups. *P. aeruginosa* infection, compared to the PBS/PBS group, induced significant bacterial dissemination measured by spleen bacterial load. Moreover, bacterial dissemination was significantly decreased for the MNV/CHA group compared to the PBS/CHA group (Figure 3).

### Murine norovirus infection modulates *P. aeruginosa* induced pro-inflammatory response

**Figure 4:**
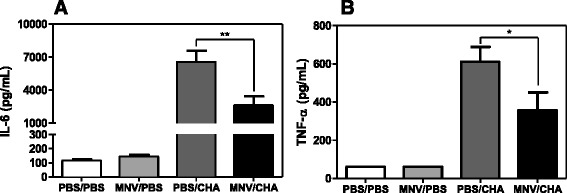
**Pro-inflammatory response.** WT mice were infected *per os* with 1.10^7^ PFU of MNV or PBS (*n* = 10/group). A week later, mice were infected with intranasal instillation of 2.10^6^ CFU of *P. aeruginosa* or PBS. **A** IL-6 and **B** TNFα protein were measured by ELISA in supernatants of BAL. *, *P* < 0.05; **, *P* < 0.01; ***, *P* < 0.001.

We finally analysed the synthesis of TNF alpha and interleukin 6 under the same experimental conditions. No difference in cytokine production was observed between the two control groups PBS/PBS and MNV/PBS. However, the production of these two pro-inflammatory cytokine in response to acute infection was significantly reduced in mice pre exposed to MNV compared to the PBS/CHA group (Figure 4).

## Discussion

Murine norovirus is frequently encountered in research mouse colonies therefore raising the question of its potential influence in mouse models. Recent data show that norovirus target immune cells. Indeed, the murine norovirus is capable of infecting macrophages, dendritic cells and B lymphocytes in vitro as well as in vivo [24-26]. There is evidence of extra-intestinal spread of MNV. The cellular tropism of murine norovirus is known for macrophages and migratory dendritic cells [5], it is possible that norovirus diffuse into the way associated with the cells of organism. This is supported by a recent study in which the depletion of mouse dendritic cells prevents the spread of murine norovirus in mesenteric lymph nodes [27].

MNV displays tropism for myeloid cells and establishes persistent infection without causing obvious disease in immune-competent mice [25]. This tropism for myeloid cells suggests a potential effect on inflammation and the immune responses. For the past few years, most of the studies focused on the interference of MNV on biomedical mouse models of inflammatory bowel disease (IBD). For example, ATG16L1 mice infected with MNV CR6 exhibited multiple hallmarks of human Crohn’s disease after dextran sodium sulfate administration compared to mice infected with MNV-1 CW3 [28]. In addition, the observed phenotype is dependent on the timing of infection. In fact, inflammatory hallmarks of Crohn’s disease are only observed when a lap of time of 7 days between viral infection and DSS protocol are respected and not when viral infection and DSS protocol are experimented at the same time.

Despite the large number of published studies on MNV, little is known regarding consequences of MNV in infectious models other than the gastrointestinal tract target. Globally, infection in mice is not dramatically altered by MNV. In fact, MNV CR6 did not alter immune responses in C57BL/6 mice co-infected with Friend virus [29], influenza A virus [14], vaccinia virus [14] or murine cytomegalovirus [30]. The effect of murine norovirus 4 Ldlr KO mouse model of atherosclerosis was found to be dependent on the time of infection [31,32]. Therefore, the analysis of all of these studies highlights the importance of different variables. Indeed, the observed phenotypes are dependent on the MNV strain, murine genetic background, and the timing of infection used in the study.

Since the late twentieth century, infectious diseases rarely affect immunocompetent hosts. The emergence of these diseases that do not infect healthy individuals shows that existing pathogenicity and virulence concepts do not include the host contribution as much as the microorganism in microbial pathogenesis and the resulting symptoms. To remedy this problem, a new theoretical approach to the understanding of microbial pathogenesis known as the “damage-response” was proposed by Casadevall and Pirofski [33]. This theory is based on three principles: the pathogenesis of microbial infections is due to the interaction between the host and the microorganism; the host prognosis is determined by the severity of tissue damage from the host pathogen interaction; induced tissue damage is due to both the pathogen but also the response of the host. Neutrophils play a key role in bacterial clearance but are also implicated in the post-acute lung injury to a bacterial infection. Indeed, the severity of these lesions is dependent on the intensity and duration of the inflammatory process. The role of pro-inflammatory molecules such as TNF-α in bacterial infection is complex contributing to host defence, but also participating in organ damage and lethality [34]. For example, Kim et al showed that infection with MNV-1 increased lethality induced by secondary infection with *E. coli*, and this was mediated, in part, by TNF-α production [18]. Interestingly, we showed that MNV S99 could modulate the immune response in acute lung infection and decrease mortality. In our previous work the inoculum was comparable but the bacterial infection was performed only 24 h after the viral infection. Moreover the route for the bacterial infection was also different (intraperitoneal versus intranasal). The main difference is probably related to the timing between the viral and bacterial infection. Recently Kernbauer et al. have shown a protective effect of CR6 and Ski strains of murine norovirus. Indeed, the administration of these strains has improved the survival of mice treated with a cocktail of antibiotics subjected to chemical colitis protocol. In addition, the authors demonstrated that MNV viral infection could provide protection against intestinal lesions induced by *C. rodentium* [35].

To our knowledge, this is the first report directly testing the effect of MNV S99 on a mouse model of acute lung infection. This study demonstrates unequivocally that MNV modulates the response to *Pseudomonas aeruginosa* induced lung injury. The S99 strain of murine norovirus led to a reduction in the inflammatory response that is found exaggerated and harmful for mice only infected with the CHA strain of *P. aeruginosa*. Studies have demonstrated that the MNV could disseminate in the organism through the cells it infects. Therefore, the MNV can be found in the lungs and diverting the cellular machinery of the immune cells for replication thereby reducing the immune response. Also, this virus can function in a manner analogous to commensal bacteria and the presence of certain virus strains to be beneficial for the host.

MNV is a prevalent and endemic virus in research mouse colonies. Our studies suggest that MNV infection can alter the survival and the immune response in a mouse model for acute lung infection. Given the prevalence of MNV in animal facility research, it is now necessary to consider this unwelcomed parameter.
